# A Supramolecular Sensing Platform for Phosphate Anions and an Anthrax Biomarker in a Microfluidic Device

**DOI:** 10.3390/ijms12117335

**Published:** 2011-10-26

**Authors:** Bilge Eker, Mahmut Deniz Yilmaz, Stefan Schlautmann, Johannes G. E. Gardeniers, Jurriaan Huskens

**Affiliations:** 1 Mesoscale Chemical Systems, MESA+ Institute for Nanotechnology, University of Twente, 7500 AE, Enschede, The Netherlands; E-Mails: b.eker@utwente.nl (B.E.); s.schlautmann@utwente.nl (S.S.); 2 Molecular Nanofabrication Group, MESA+ Institute for Nanotechnology, University of Twente, 7500 AE, Enschede, The Netherlands; E-Mail: m.d.yilmaz@utwente.nl (M.D.Y.)

**Keywords:** microfluidic sensing device, phosphate and anthrax biomarker detection, lanthanide-based supramolecular sensing, surface-assisted ATP and DPA detection

## Abstract

A supramolecular platform based on self-assembled monolayers (SAMs) has been implemented in a microfluidic device. The system has been applied for the sensing of two different analyte types: biologically relevant phosphate anions and aromatic carboxylic acids, which are important for anthrax detection. A Eu(III)-EDTA complex was bound to β-cyclodextrin monolayers via orthogonal supramolecular host-guest interactions. The self-assembly of the Eu(III)-EDTA conjugate and naphthalene β-diketone as an antenna resulted in the formation of a highly luminescent lanthanide complex on the microchannel surface. Detection of different phosphate anions and aromatic carboxylic acids was demonstrated by monitoring the decrease in red emission following displacement of the antenna by the analyte. Among these analytes, adenosine triphosphate (ATP) and pyrophosphate, as well as dipicolinic acid (DPA) which is a biomarker for anthrax, showed a strong response. Parallel fabrication of five sensing SAMs in a single multichannel chip was performed, as a first demonstration of phosphate and carboxylic acid screening in a multiplexed format that allows a general detection platform for both analyte systems in a single test run with μM and nM detection sensitivity for ATP and DPA, respectively.

## 1. Introduction

It is of utmost importance to detect low concentrations of small molecules and solutes in mixtures of complex milieu for toxicology [1–3], drug discovery [4–6], diagnostics [7–9], and antibioterrorism [10,11]. Among many chemical, electrochemical, biological, and photoluminescence based small molecule detection, fluorescence turn-on/off sensing has attracted significant attention, offering high sensitivity [12–15] and reversibility [16,17]. Lanthanide (Ln^3+^) based luminescent detection for small molecules such as nerve agents [1], antagonists [4], phosphate anions [18,19], and the anthrax biomarker DPA [20–22] has been most promising among the many turn-on/off sensing methods owing to the unique photophysical properties of Ln^3+^-antenna chelates with their bright luminescence upon sensitization by an antenna, long luminescence lifetimes compared to free Ln^3+^, and sharp line-like emission bands at long wavelengths [23–25], overcoming autofluorescence and light scattering, and resulting in a high color purity of the emitted light [26].

Surface-confined sensing using self-assembled monolayers (SAMs) offers advantages compared to sensing in solution such as ease and reproducibility of SAMs [27,28], fast response times owing to the faster analyte-receptor interaction, and real-time and real-space measurements [29,30]. Previous studies have shown that SAMs can function as optical sensors when functionalized with fluorescent groups on flat surfaces [31–33] and in microfluidic systems [34,35].

Phosphate anions play an important role in various physiological events and they take part in almost all metabolic processes. Among various phosphates, pyrophosphate and adenosine triphosphate (ATP) are crucial anions for the transfer of genetic information, and metabolic and bioenergetic reactions [36]. Fluorescence based detection of biologically relevant phosphate anions still remains a challenge due to the difficulty of designing binding motifs for anions [37] and achieving an effective fluorescent signal change upon anion binding [15,38]. Recently, a Ln^3+^-based displacement assay was employed for phosphate anions on gold nanoparticles in solution [19] and on a gold surface [18]. However, to the best of our knowledge, the combination of Ln^3+^ luminescence based detection of phosphate anions and surface-based sensing on macro and micro scale glass and in particular in a glass/silicon microfluidic channel has not been achieved to date.

Concurrently, some aromatic carboxylic acids have crucial importance in bioterrorism, especially in anthrax detection [39]. Anthrax is an acute disease and a potential biological warfare agent caused by *Bacillus Anthracis*. The detection of *Bacillus* spores with rapid response, high selectivity, and sensitivity is very important in order to minimize the impact of a bioterroristic attack or outbreak of disease [40–42]. *Bacillus* spores contain up to 1 M dipicolinic acid (DPA), corresponding to 5–15% of the dry mass of the bacterial spore [43–45], offering a convenient biomarker for anthrax detection. Ln^3+^-based luminescent detection of DPA has been very promising by using DPA as an antenna itself [20,21,46] or as Ln^3+^-DPA chelates [22], allowing fast, highly selective and sensitive detection of bacterial spores. Recently, ratiometric detection of DPA was employed by fabricating microarrays via patterning of a monolayer surface [22]. However, the integration of surface-based DPA sensing with microfluidics has not been performed yet.

In the current study we present a novel multiplexed platform for a general detection method of biologically relevant phosphate anions and the *Bacillus Anthracis* biomarker DPA on a supramolecular monolayer surface by using a microfluidic approach. The microfluidic approach has attracted significant attention in the last decades for chemical and biological assays because of faster detection time, low consumption of analyte and reagents, and the possibility of integrated continuous monitoring of analyte solutions [35,47–50]. Here, β-cyclodextrin monolayers known as molecular printboards [51] were employed on a glass-silicon microchip surface and building blocks were attached to the monolayers in a noncovalent fashion that allow phosphate anion and DPA sensing. To the best of our knowledge, this microchip-based sensing platform constitutes the first lanthanide-based surface receptor system that provides a general detection platform for two different analyte systems, as well as the first example of integration of sensing of phosphate anions and DPA in a microfluidic device.

## 2. Results and Discussion

### 2.1. Fabrication of the Sensing Platform and Anion Detection

Surface-assisted sensitized luminescence of Eu^3+^ on a molecular printboard was demonstrated in a previous study for the sensing of biological molecules such as the *Bacillus Anthracis* biomarker DPA [22]. In the current study, luminescent SAMs on a microchip surface were fabricated to develop a general detection platform of small molecules such as the biologically relevant phosphate anions and aromatic carboxylic acids in a multiplexed format. The sensing platform was developed in a microfluidic device by using the controlled attachment of the building blocks in a supramolecular manner, through multivalent orthogonal linkers. Such a supramolecular surface platform was chosen for this study, since supramolecular interactions combined with multivalency offer high flexibility such as controlled positioning of molecules, fine-tuning of assemblies and their interaction properties, binding strength, binding stoichiometry, binding dynamics, and reversibility [51]. β-Cyclodextrin (βCD)-based host-guest chemistry at the microchip interface was applied to orient the attachment of building blocks of interest to the microchip surface. Microchannels are miniaturized platforms that can be individually accessible and addressable, allowing production of sensing arrays for multianalyte systems within a single device. Thereby, the supramolecular sensing system was implemented on the surface of a multichannel chip as a demonstration of a high-throughput sensing device.

**Figure 1:**
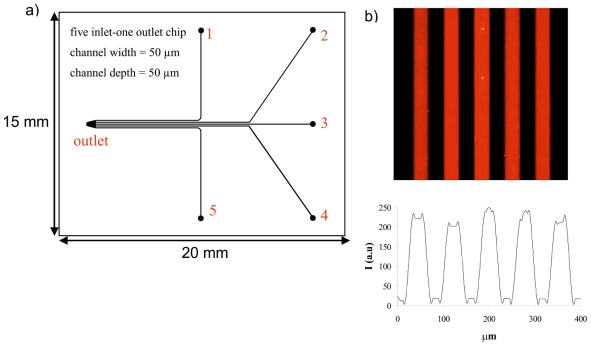
(**a**) Design of the five inlet-one outlet multichannel chip surface; (**b**) A fluorescence microscopy image and concomitant intensity profile of the red luminescent multichannel device.

**Scheme 1:**
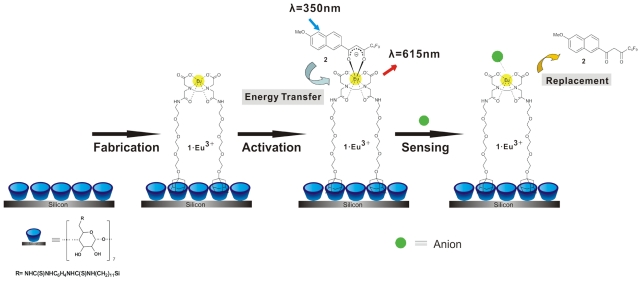
The construction of supramolecular sensing system and the detection of an anion of interest.

A multichannel chip with five parallel channels was used for generation of the sensing array via the formation of βCD SAMs and subsequent attachment of building blocks of interest on each channel surface (Figure 1a). Parallel synthesis of five sensing SAMs in the multichannel chip was performed by the formation of βCD SAMs on the channel surface as described above, leading to an array of five βCD SAMs confined to a single chip, and providing a functional layer for further surface modification in the multichannel. Two building blocks were used to fabricate the sensor surface in the microchip: an ethylenediamine tetraacetic acid (EDTA)-based ligand (**1**) for binding Eu^3+^ and the receptor surface, and a naphthalene-based antenna (**2**) for coordination to Eu^3+^ via the diketonate moiety (Scheme 1). Building block **1** has adamantyl groups for immobilization onto the βCD SAMs. To fabricate sensing surfaces on the microchip surface, a stepwise procedure was followed. Briefly, in the first step, 1 mM of **1.**Eu^3+^ was attached on the βCD monolayer after 30 min incubation in the microchip. After cleaning the microchip surface with water for 10 min, 100 μM of **2** was incorporated onto βCD SAMs via coordination of **2** with **1.**Eu^3+^ as seen in Scheme 1. After a second cleaning step of the microchip surface with water for 10 min, the multichannel surface was imaged by fluorescence microscope using filter R (300 nm < *λ_exc_*< 400 nm, *λ_em_* = 615 nm). Five luminescent SAM-modified microchannels were visualized simultaneously (Figure 1b) by fluorescence microscopy, which showed energy transfer from the Eu-coordinated napthalene moiety of **2** to the Eu^3+^ center of **1**.Eu^3+^, and the emission of red light at 615 nm as studied before [22,45]. Thereby, a highly luminescent surface platform was achieved in the multichannel device for the detection of phosphates and aromatic carboxylic acids.

The mechanism of anion detection is based on the displacement of **2** from **2**-**1**.Eu^3+^ by a guest anion as shown in Scheme 1. Displacement assays have been studied before and the mechanism of displacement is well established [17,52,53] and the same mechanism is assumed in this study. Briefly, the sensing layer on the microchip surface is an ensemble of **1**.Eu^3+^ and the antenna **2**, and when an anionic guest is added to the ensemble, it displaces **2**, and triggers a fluorescence change upon binding to the **1**.Eu^3+^, leading to a decrease in red fluorescence at 615 nm. Thus, the detection of phosphates and aromatic carboxylic acids is based on the recognition of the guest anion by the Eu^3+^-based receptor and the displacement of **2**. Thus, the resulting decrease in the fluorescence intensity was monitored and quantified by fluorescence microscopy.

### 2.2. Sensing of Biologically Relevant Phosphates

**Figure 2:**
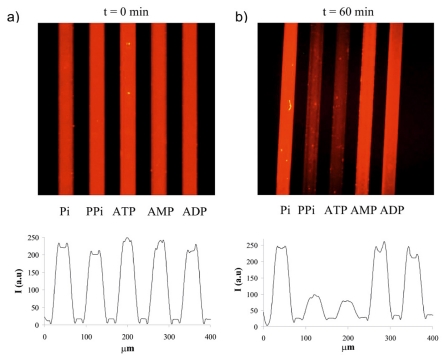
(**a**) The effect of phosphate anions at 1 mM concentration each (from left to right; hydrogen phosphate (Pi), pyrophosphate (PPi), adenosine triphosphate (ATP), adenosine monophosphate (AMP), adenosine diphosphate (ADP)) on the displacement of the antenna from **2**-**1**.Eu^3+^ on the multichannel surface after 0 min; (**b**) 60 min, using a flow rate of 1 μL/min.

**Figure 3:**
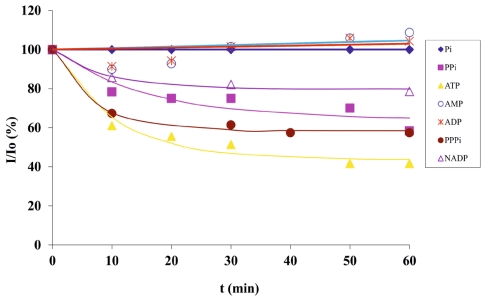
Fluorescence intensity *vs*. time profiles of 1 mM phosphates (Pi, PPi, ATP, AMP, ADP, PPPi, NADP) for selectivity and response time determination on a microchannel surface.

The luminescent multichannel platform was used for the detection of biologically relevant phosphate anions. Five different phosphates (1 mM each), ATP, adenosine monophosphate (AMP), adenosine diphosphate (ADP), hydrogen phosphate (Pi) and pyrophosphate (PPi) were injected continuously from each inlet (at 1 μL/min by a peristaltic pump) and their sensing was evaluated within 1 h. Among those five phosphate anions, only ATP and PPi caused a strong response at the **2**-**1**.Eu^3+^ complex (Figure 2a,b). The sensing of these five anions along with nicotinamide adenine dinucleotide phosphate (NADP) and triphosphate (PPPi) was also studied as a function of time, and around 40% and 35% decrease in fluorescence intensity was observed for both ATP and PPPi, while 22% and 15% quenching of red emission was observed for PPi, and NADP, respectively, in 10 min (ATP > PPPi > PPi > NADP at *t* = 10 min) as seen in Figure 3. Around 60% decrease in fluorescence intensity was obtained for ATP after 1 h, whereas PPPi and PPi showed around 45% decrease in red emission, and the effect of NADP on displacement of the antenna was around 20% after 1 h (ATP > PPPi ≈ PPi > NADP at *t* = 60 min). The response half-time of ATP was determined based on the exponential decay curve in Figure 3, and was found to be about 10 min.

The different sensing properties of the anions on the microchannel surface are presumably due to the different binding affinity of the anions to Eu^3+^, which is correlated with number of phosphate oxygens that coordinate to Eu^3+^ after the displacement of the antenna [54]. As seen in Figure 3, Pi, AMP and ADP showed no response (similar to only buffer, data not shown). In contrast, PPPi displaced **2** almost as effective as ATP indicating the importance of the number of phosphate oxygens on the strong coordination with Eu^3+^ [55,56] resulting in displacement of **2** from **2**-**1**.Eu^3+^. PPi as a diphosphate also caused a fairly strong response of the **2**-**1**.Eu^3+^ complex owing to its high binding affinity to Eu^3+^ [54]. On the other hand, among the diphosphates PPi, ADP, and NADP different responses were seen, especially the sensing of PPi was much stronger than the other diphosphates indicating that the side groups of ADP and NADP might decrease the binding affinity of the phosphate groups to Eu^3+^, thus leading to less effective displacement of **2** from **2**-**1**.Eu^3+^ compared to PPi. Thus, these results show that the phosphates possess different sensitivities to the **2**-**1**.Eu^3+^ complex, especially ATP, PPi and PPPi exhibit stronger response to the Eu^3+^ based assembly compared to the other phosphates.

**Figure 4:**
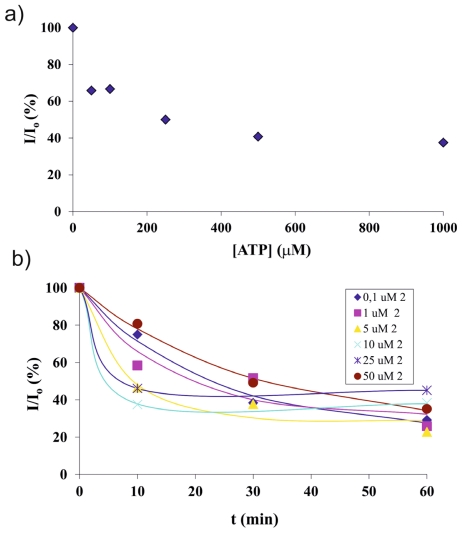
(**a**) The detection sensitivity profile of ATP at 60 min as a function of different ATP concentrations; (**b**) Fluorescence intensity *vs*. time profiles of 1 mM ATP in the presence of different concentrations of **2**.

To determine the detection limit of ATP sensing on the microchip platform, ATP detection was performed as a function of concentration. Five different concentrations ranging from 1 mM down to 50 μM ATP were injected continuously from each inlet by a peristaltic pump and their sensing was evaluated within 1 h. Figure 4a shows the normalized intensity (I/I_0_) profile of five different concentrations at *t* = 60 min. Similar responses were observed with ATP concentrations from 1 mM down to 250 μM (around 50%–60%), and around 35% decrease in red emission was obtained for 50 μM ATP (Figure 4b). Thus, ATP sensing by the Eu^3+^ based complex was achieved in the μM range. This is consistent with earlier reported data, which showed ATP detection in the μM [36,57,58] or mM concentration range [59].

The experiments described above were all performed in the absence of **2** in solution, therefore, displacement of **2** led to removal of **2** from the system and thus to kinetically determined fluorescence readings. Subsequently, ATP sensing was performed in the presence of different concentrations of **2** in order to investigate ATP detection while attempting to reach thermodynamic equilibrium of the displacement reaction of **2** from **2**-**1**.Eu^3+^ by ATP. Mixtures of 1 mM ATP with five different concentrations of **2**, ranging from 0.1 μM to 50 μM, were injected continuously from each inlet and their sensing of **2**-**1**.Eu^3+^ was evaluated within 1 h. Figure 4b shows the decrease in red fluorescence was similar for all conditions. No clear trend was obtained for different concentrations of **2**, nor in response time. These results show that the binding strength of ATP to Eu^3+^ is much higher than that of the antenna **2**.

### 2.3. Screening of an Anthrax Biomarker and Potentially Interfering Anions

**Figure 5:**
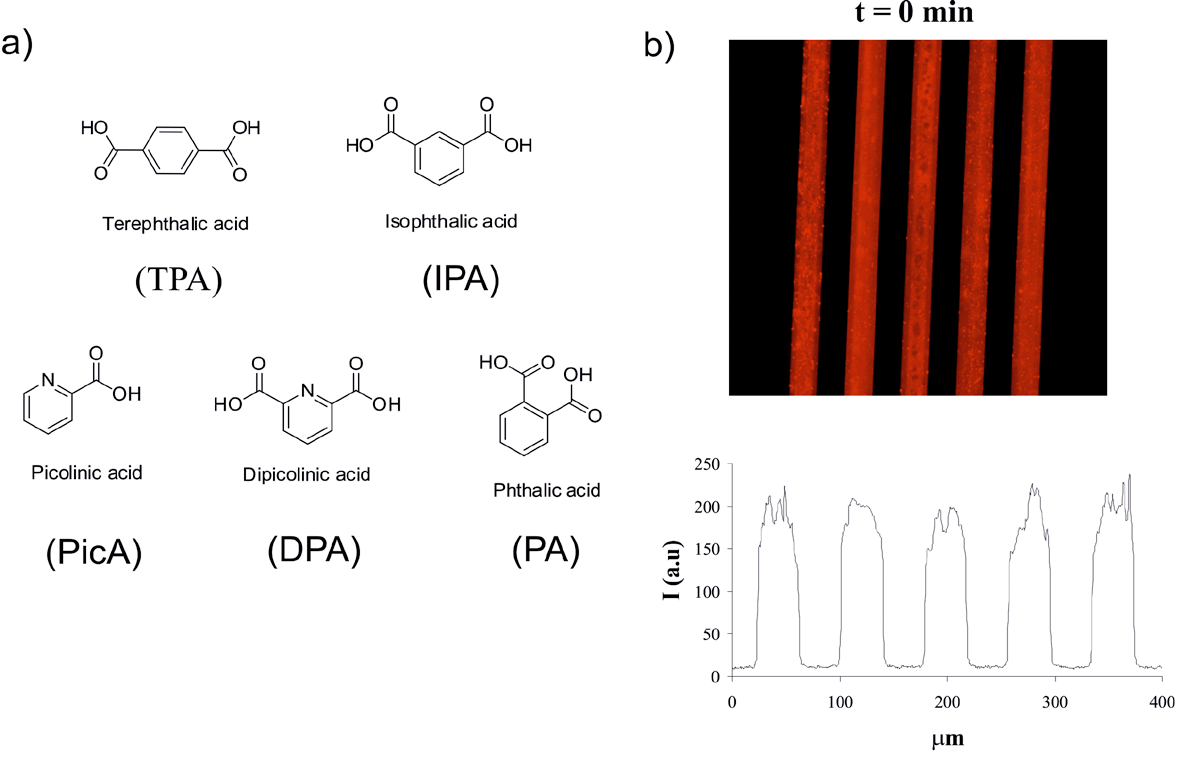

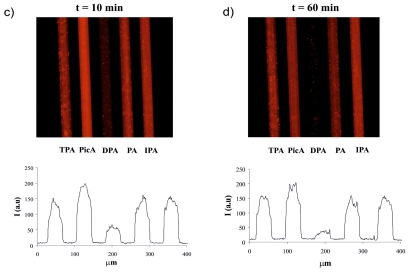
(**a**) The structure of aromatic carboxylic acids: *p*-phthalic acid (TPA), picolinic acid (PicA), dipicolinic acid (DPA), *o*-phthalic acid (PA), and *m*-phthalic acid (IPA); (**b**) Fluorescence microscopy images and intensity profiles of the multichannel surface upon carboxylic acid screening at *t* = 0 min; (**c**) *t* = 10 min; (**d**) and *t* = 60 min.

**Figure 6:**
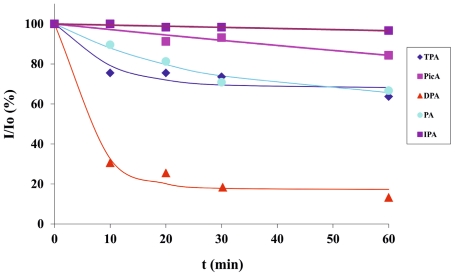
Fluorescence Intensity *vs.* time profiles of 1 mM aromatic carboxylic acids (*p*-phthalic acid, picolinic acid, DPA, *o*-phthalic acid, and *m*-phthalic acid)) for selectivity and response time determination on the microchannel surface.

The same supramolecular lanthanide-based microfluidic platform was also used for the detection of the anthrax biomarker DPA, among other potentially competitive aromatic ligands as a demonstration of a surface-based sensing device that offers a general high-throughput detection platform both for phosphates and aromatic carboxylates. In order to investigate aromatic carboxylic acid detection, five different aromatic carboxylic acids (1 mM each), *o/m/p*-phthalic acids, picolinic acid and DPA, were injected from each inlet continuously and their sensing was evaluated within 1 h. Among those five aromatic ligands (Figure 5a), only DPA was sensed by **2**-**1**.Eu^3+^ and selectively displaced the antenna, thus quenched around 80% of the red luminescence in 10 min whereas the effect of the other aromatic acids on the displacement of **2** was negligible (up to 25% quenching of red emission with terephthalic acid) (Figure 5b,c and Figure 6). Excellent selectivity was achieved with DPA over other potentially interfering aromatic acids after 1 h (Figure 5d, Figure 6), while other interferants showed weak response on the sensor surface (up to 35% quenching of red emission with picolinic and isophthalic acid). The response half-time of DPA was determined based on the exponential decay curve in Figure 6, and DPA showed a response half-time of around 5 min.

The high selectivity of DPA sensing on **2**-**1**.Eu^3+^ is attributed to a higher binding affinity of DPA to Eu^3+^ [60,61] due to its stronger coordination to Eu^3+^ via two carboxylic acid groups and the basic nitrogen atom on its aromatic ring, whereas the other aromatic ligands coordinate to Eu^3+^ via fewer groups. In addition, the carboxylic acid screening in solution was also performed and only DPA selectively displaced **2** after 10 min (data not shown) owing to the much stronger coordination of DPA with **1**.Eu^3+^ than that of **2**, showing consistent results with those obtained on receptor surface. Thus, these results show that all aromatic carboxylic acids show different sensing properties to **2**-**1**.Eu^3+^ complex, and especially DPA exhibited excellent selectivity and a quick response time (within 10 min). Thus, this multichannel sensing platform offers a common detection system for both aromatic carboxylic acids and phosphates.

**Figure 7:**
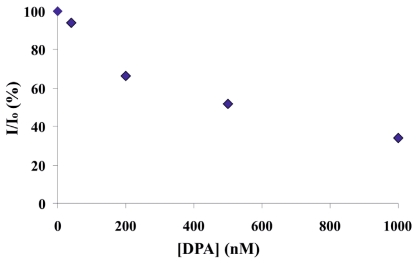
The detection sensitivity profile of DPA at 60 min as a function of different DPA concentrations.

To determine the detection limit of DPA sensing on the microchip platform, DPA detection was performed as a function of concentration. Initially, five different concentrations ranging from 1 mM down to 500 nM DPA were injected continuously from each inlet by a peristaltic pump and their sensing was evaluated within 1 h. Lower concentrations down to 40 nM were also used to determine the detection limit of DPA. The intensity ratio (I/I_0_) values of the sensor substrates were plotted as a function of the DPA concentration (0–1 μM) at *t* = 60 min in Figure 7. Lower concentrations of DPA resulted in less quenching of the red emission showing that DPA sensing is concentration dependent. 1 μM DPA quenched red emission as effective as 1 mM DPA (around 70%–80%), and nM concentrations of DPA still showed significant response on the sensor surface (Figure 7). Thus, DPA was sensed by the Eu^3+^ based complex with <100 nM detection limit. Such a detection sensitivity range is consistent with literature [22].

In a recent study, ratiometric detection of DPA was employed by us at molecular printboards using a lanthanide-based surface-receptor system [22]. A different naphthalene based antenna with a carboxylic acid side chain was used for the sensing platform, and carboxylic acid sensing was performed on glass substrates via microcontact printing method. DPA showed high selectivity over other potentially competitive aromatic ligands in water with nanomolar sensitivity, and the sensing response was complete within 10 min on glass substrate, showing consistent and similar results to those reported here. However, that surface receptor system was only used for DPA sensing, and phosphates did not show any response to the sensing molecular printboards. When different antenna **2** was used for this study to develop the sensing monolayer on the microchannel surface**,** we have achieved to sense both carboxylic acids and also phosphates on the same surface-based sensing platform. In addition, evaporation of naphthalene on a glass substrate resulted in precipitation of the antenna on the surface leading to nonuniform sensing platform, and complicated the visualization during the analysis. Integration of microfluidics with such a sensing surface eliminates evaporation problems owing to the enclosed microchannels, and continuous naphthalene perfusion during the surface modification. Uniform sensing surface with high red fluorescence was obtained on the microchannels, resulting in continuous monitoring of sensing surface after anion injection. Moreover, the core of the bacterial spores contains up to 1 M DPA [43–45], which is ~7 orders of magnitude higher than our detection limit (~200 nM). A practical real-world application might involve releasing DPA from the aerosolized bacterial spores into bulk solution by microwaving or autoclaving the sample [11,62], and detecting the released DPA in aqueous solution by using the displacement assay on the microchip surface in continuous, label-free and real-time mode. The decrease in the luminescence intensity can be correlated to DPA concentration, and subsequently to bacterial spore concentration. Thereby, our study demonstrates the development of a microfluidic device for the first time based on displacement strategy by using a different antenna **2**, which allows the detection of bacterial spores along with other type of anions such as biologically relevant phosphates at a multiplexed format.

## 3. Experimental Section

### 3.1. Compounds

Unless otherwise noted, solvents and reagents were reagent grade and used without further purification. β-Cyclodextrin (βCD) heptamine, the Eu(III)-EDTA complex **1**.Eu^3+^ and the antenna naphthalene β-diketone **2** were synthesized as described before [63–65].

### 3.2. Microchip Fabrication Procedure

The silicon-glass microchip was fabricated by following standard microfabrication processes [66]. Microchannels were defined in a (100) silicon wafer (resistivity 5–10 Ω cm, diameter 100 mm) via standard UV-lithography using a mask of photoresist (Olin 907-17 photoresist) followed by deep reactive ion etching (BOSCH-type process). Inlet and outlet holes were fabricated by powderblasting [67]. The silicon wafer was bonded to Borofloat glass via anodic bonding (*T* = 400 °C, U_max_ = 1200 V, *t* = 10 min) and the silicon glass wafer was diced into separate chips (chips size: 15 mm × 20 mm). Channel dimensions were 50 μm × 50 μm (width × depth). The length of a single channel of interest was 6 mm and separation between the channels was 50 μm.

### 3.3. Surface Modification of the Microchips

The microchannel surface was functionalized with βCD SAMs by following a three-step reaction, as described by Ludden *et al*. [68]. Prior to surface modification, intense piranha cleaning was performed inside the microchip via vacuum and then followed by water cleaning. After drying the chip in a stream of N_2_, approximately 500 μL freshly distilled toluene was flushed through the chip. Thereafter, a 5 mM solution of N-[3-(trimethoxysilyl)propyl]ethylenediamine (TPEDA) in toluene was injected through the microchip by the syringe pump with a 0.1 μL/min flow rate for 4 h at room temperature. Distilled toluene was flushed through the chip for 30 min at a flow rate of 0.1 μL/min and then followed by flushing at 10 μL/min for 5 min to get rid of any possible aggregates from the channels. After this step, 10 mM 1,4-phenylene-diisothiocyanate (DITC) in toluene was flushed inside the microchip and incubated for 4 h at 60 °C followed by a rinse with toluene and then with ethanol. After drying the chip, the chip was washed with Millipore water for 5 min, and 10 mM βCD heptamine in Millipore water was injected and incubated in the chip for 2 h at 60 °C, followed by a rinse of the chip with Millipore water for 5 min.

### 3.4. Assembly of the Sensing Layer on the Microchip Surface

The Eu(III)-EDTA complex was assembled onto the βCD monolayers by incubation of 1 mM **1**.Eu^3+^ in water in the microchip for 30 min. This results in immobilization of the Eu(III)-EDTA complex onto the βCD monolayers via orthogonal supramolecular host-guest interactions. Thereafter, 100 μM of the antenna naphthalene β-diketone in water was injected and incubated for 30 min in the microchip. The self-assembly of the Eu(III)-EDTA conjugate and naphthalene β-diketone resulted in the formation of a highly red luminescent lanthanide system on the microchannel surface via the energy transfer from the antenna to the surface-anchored Eu(III).

### 3.5. Microfluidic Fluorescent Sensing Experiments

Different analytes (phosphates such as ATP, ADP, AMP, pyrophosphate, hydrogen phosphate and carboxylic acids such as *o/m/p*-phthalic acids, picolinic acid and dipicolinic acid) in 50 mM HEPES buffer with 50 mM NaCl (pH 7.4) were injected from five inlets with a 1 μL/min flow rate at room temperature at a multiplexed format for turn-off sensing experiments. For these sensing experiments, all phosphates and carboxylic acids were used at 1 mM concentration. For detection sensitivity experiments, an ATP concentration down to 50 μM and a DPA concentration down to 40 nM was used. The decrease in red emission for each channel in the microchip upon sensing was quantified using a fluorescence microscope using filter R (*λ_em_* = 615 nm), and a narrow band excitation filter (300 nm < *λ_exc_* < 400 nm) was used to eliminate direct excitation of analytes such as DPA (*λ_exc_* = 270 nm). The data shown in Figures 3 and 6 were reproduced 5 times on the same chips, and found to be reproducible to within 10%.

## 4. Conclusions

In conclusion, we have developed a supramolecular sensing platform on a microchip surface that allows the detection of biologically relevant phosphate anions and aromatic carboxylic acids at a multiplexed format. ATP and pyrophosphate among various phosphate anions, and the anthrax biomarker DPA among various aromatic carboxylic acids, showed a strong response to the sensitized Eu^3+^ luminescence-based microchip surface, exhibited by strong quenching of red emission for the anions and DPA in a few to tens of minutes. ATP was sensed by **2**-**1**.Eu^3+^ and showed μM detection sensitivity. Higher detection sensitivity (~100 nM) was achieved for DPA.

Therefore, as a first demonstration of phosphate and carboxylic acid sensing on the same lanthanide-based surface-receptor platform in a microfluidic chip at a multiplexed format, this study offers a general detection platform for phosphates and carboxylic acids in a single test run with high detection sensitivity and selectivity. From this point of view, this supramolecular surface assisted sensing system in multichannel opens new avenues to yield new classes of surface-based detection devices for biologically relevant ions and bacterial spores. Real-time imaging and continuous monitoring of analyte solutions with the power of microfluidics in combination with creating functional systems with the power of supramolecular chemistry offers new designs of miniaturized sensing systems for different kinds of small molecules, which might have biological and diagnostic importance. Overall, this system exemplifies the significance of microfluidics and noncovalent strategies to create microscale sensing devices by fine-tuning different small building blocks, which provide different functionalities when assembled onto such a sensor surface.

## References

[1] Jenkins A.L., Uy O.M., Murray G.M. (1999). Polymer-Based Lanthanide Luminescent Sensor for Detection of the Hydrolysis Product of the Nerve Agent Soman in Water. Anal. Chem.

[2] Puopolo P.R., Chamberlin P., Flood J.G. (1992). Detection and confirmation of cocaine and cocaethylene in serum emergency toxicology specimens. Clin. Chem.

[3] van der Schalie W.H, Shedd T.R., Widder M.W., Brennan L.M. (2004). Response characteristics of an aquatic biomonitor used for rapid toxicity detection. J. Appl. Toxicol..

[4] Rega M.F., Reed J.C., Pellecchia M. (2007). Robust lanthanide-based assays for the detection of anti-apoptotic Bcl-2-family protein antagonists. Bioorg. Chem.

[5] Hajduk P.J., Gerfin T., Boehlen J.M., Haberli M., Marek D., Fesik S.W. (1999). High-Throughput Nuclear Magnetic Resonance-Based Screening. J. Med. Chem.

[6] Huestis M.A, Smith ML (2006). Modern analytical technologies for the detection of drug abuse and doping. Drug Discov..

[7] Fu E., Chinowsky T., Nelson K., Johnston K., Edwards T., Helton K., Grow M., Miller J.W., Yager P. (2007). SPR imaging-based salivary diagnostics system for the detection of small molecule analytes. Ann. NY Acad. Sci.

[8] Smith A.M., Dave S., Nie S., True L., Gao X. (2006). Multicolor quantum dots for molecular diagnostics of cancer. Expert Rev. Mol. Diagn.

[9] Haas P., Then P., Wild A., Grange W., Zorman S., Hegner M., Calame M., Aebi U., Flammer J., Hecht B. (2010). Fast quantitative single-molecule detection at ultralow concentrations. Anal. Chem.

[10] Lester E., Ponce A (2002). An anthrax “smoke” detector. Online monitoring of aerosolized bacterial spores. IEEE Eng. Med. Biol.

[11] Lester E., Ponce A (2004). A second-generation anthrax “smoke” detector. IEEE Eng. Med. Biol.

[12] Lakowicz J.R. (1999). Principles of Fluorescence Spectroscopy.

[13] Narayanaswamy R., Wolfbeis O.S. (2004). Springer Series on Chemical Sensors and Biosensors.

[14] Martinez–Manez R., Sancenon F. (2005). New advances in fluorogenic anion chemosensors. J. Fluoresc.

[15] Martinez–Manez R., Sancenon F. (2003). Fluorogenic and chromogenic chemosensors and reagents for anions. Chem. Rev.

[16] Niikura K., Metzger A., Anslyn E.V. (1998). Chemosensor ensemble with selectivity for inositol-trisphosphate. J. Am. Chem. Soc.

[17] Metzger A., Anslyn E.V. (1998). A Chemosensor for Citrate in Beverages. Chem. Int. Ed.

[18] Murray N.S., Jarvis S.P., Gunnlaugsson T. (2009). Luminescent self-assembly formation on a gold surface observed by reversible “off–on” switching of Eu(III) emission. Chem. Commun.

[19] Massue J., Quinn S.J., Gunnlaugsson T. (2008). Lanthanide luminescent displacement assays: the sensing of phosphate anions using Eu(III)-cyclen-conjugated gold nanoparticles in aqueous solution. J. Am. Chem. Soc.

[20] Cable M.L., Kirby J.P., Sorasaenee K., Gray H.B., Ponce A. (2007). bacterial spore detection by [Tb^3+^(macrocycle)(dipicolinate)] luminescence. J. Am. Chem. Soc.

[21] Ai K., Zhang B., Lu L. (2009). Europium-Based fluorescence nanoparticle sensor for rapid and ultrasensitive detection of an anthrax biomarker. Angew. Chem. Int. Ed.

[22] Yilmaz M.D., Hsu S., Reinhoudt D.N., Velders A.H., Huskens J. (2010). Ratiometric fluorescent detection of an anthrax biomarker at molecular printboards. Angew. Chem. Int. Ed.

[23] Leonard J.P., Nolan C.B., Stomeo F., Gunnlaugsson T. (2007). Photochemistry and photophysics of coordination compounds: lanthanides. Top. Curr. Chem.

[24] Leonard J.P., Jensen P., McCabe T., O’Brien J.E., Peacock R.D., Kruger P.E., Gunnlaugsson T. (2007). Self-assembly of chiral luminescent lanthanide coordination bundles. J. Am. Chem. Soc.

[25] Gunnlaugsson T., Stomeo F. (2007). Recent advances in the formation of luminescent lanthanide architectures and self-assemblies from structurally defined ligands. Org. Biomol. Chem.

[26] Binnemans K. (2009). Lanthanide-Based Luminescent Hybrid Materials. Chem. Rev.

[27] Crooks R.M., Ricco A.J. (1998). New Organic Materials Suitable for Use in Chemical Sensor Arrays. Acc. Chem. Res.

[28] Kaifer A.E. (1996). Functionalized Self-Assembled Monolayers Containing Preformed Binding Sites. Isr. J. Chem.

[29] Zimmerman R., Basabe–Desmonts L., van der Baan F., Reinhoudt D.N., Crego-Calama M. (2005). A combinatorial approach to surface-confined cation sensors in water. J. Mater. Chem.

[30] Kim Y.R., Kim H.J., Kim J.S., Kim H. (2008). Rhodamine-Based “Turn-On” Fluorescent chemodosimeter for Cu(II) on ultrathin platinum films as molecular switches. Adv. Mater.

[31] Flink S., van Veggel F.C.J.M., Reinhoudt D.N. (2000). Sensor functionalities in self-assembled monolayers. Adv. Mater.

[32] van der Veen N.J., Flink S., Deij M.A., Egberink R.J.M., van Veggel F.C.J.M., Reinhoudt D.N. (2000). Monolayers of a Na+ selective fluoroionophore on glass: connecting the fields of monolayers and optical detection of metal ions. J. Am. Chem. Soc.

[33] Crego-Calama M., Reinhoudt D.N. (2001). New materials for metal ion sensing by self-assembled monolayers on glass. Adv. Mater.

[34] Basabe-Desmonts L., Beld J., Zimmerman R.S., Hernando J., Mela P., García-Parajó M.F., van Hulst N.F., van den Berg A., Reinhoudt D.N., Crego-Calama M. (2004). A simple approach to sensor discovery and fabrication on self-assembled monolayers on glass. J. Am. Chem. Soc.

[35] Rudzinski C.M., Young A.M., Nocera D.G. (2002). A supramolecular microfluidic optical chemosensor. J. Am. Chem. Soc.

[36] Berg J.M., Tymoczko J.L., Stryer L (2002). Biochemistry.

[37] Ojida A., Takashima I., Kohira T., Nonaka H., Hamachi I. (2008). Turn-on fluorescence sensing of nucleside polyphosphates using a xanthene-based Zn(II) complex chemosensor. J. Am. Chem. Soc.

[38] Beer P.D., Gale P.A. (2001). Anion recognition and sensing: the state of the art and future perspectives. Angew. Chem. Int. Ed.

[39] Li Q., Dasgupta P.K., Temkin H. (2008). Airborne bacterial spore counts by terbium-enhanced luminescence detection: pitfalls and real values. Environ. Sci. Technol.

[40] Henderson D.A. (1999). The looming threat of bioterrorism. Science.

[41] Enserink M. (2001). Biodefense hampered by inadequate tests. Science.

[42] Yung P.T., Lester E.D., Bearman G., Ponce A. (2007). An automated front-end monitor for anthrax surveillance systems based on the rapid detection of airborne endospores. Biotechnol. Bioeng.

[43] Bailey G.F., Karp S., Sacks L.E. (1965). Ultraviolet-Absorption spectra of dry bacterial spores. J. Bacteriol.

[44] Walt D.R., Franz D.R. (2000). Biological warfare detection. Anal. Chem.

[45] Rode L.J., Foster J.W. (1960). Germination of bacterial spores by long-chain alkyl amines. Nature.

[46] Rosen D.L. (1999). Bacterial endospore detection using photoluminescence from terbium dipicolinate. Rev. Anal. Chem.

[47] Mela P., Onclin S., Goedbloed M.H., Levi S., García-Parajó M.F., van Hulst N.F., Ravoo B.J., Reinhoudt D.N., van den Berg A. (2005). Monolayer-functionalized microfluidics devices for optical sensing of acidity. Lab Chip.

[48] Vilkner T., Janasek D., Manz A. (2004). Micro total analysis systems. Recent developments. Anal. Chem.

[49] Andersson H., van den Berg A. (2003). Microfluidic devices for cellomics: a review. Sens. Actuator. B.

[50] Fan H.Y., Lu F.Y., Stump A., Reed S.T., Baer T., Schunk R., Perez–Luna V., Lopez G.P., Brinker C.J. (2000). Rapid prototyping of patterned functional nanostructures. Nature.

[51] Ludden M.J.W., Reinhoudt D.N., Huskens J. (2006). Molecular printboards: versatile platforms for the creation and positioning of supramolecular assemblies and materials. Chem. Soc. Rev.

[52] Metzger A., Lynch V.M., Anslyn E.V. (1997). A synthetic receptor selective for citrate. Angew. Chem. Int. Ed.

[53] Berridge M.J. (1993). Inositol trisphosphate and calcium signalling. Nature.

[54] Shao N., Jin J., Wang G., Zhang Y., Yang R., Yuan J (2008). Europium(III) complex-based luminescent sensing probes for multi-phosphate anions: modulating selectivity by ligand choice. Chem. Commun.

[55] Charbonnière L.J., Schurhammer R., Mameri S., Wipff G., Ziessel R.F. (2005). Photo-physical Impact of phosphorylated anions associated to lanthanide complexes in water. Inorg. Chem.

[56] Shanbhag S.M., Choppin G.R. (1987). Thermodynamics of Ln(III) complexation with AMP and ATP. Inorg. Chim. Acta.

[57] Kanekiyo Y., Naganawa R., Tao H (2004). Fluorescence detection of ATP based on the ATP-mediated aggregation of pyrene-appended boronic acid on a polycation. Chem Commun.

[58] Bazzicalupi C., Biagini S., Bencini A., Faggi E., Giorgi C., Matera I., Valtancoli B (2006). ATP Recognition and sensing with a phenanthroline-containing polyammonium receptor. Chem. Commun.

[59] Zyryanov G.V., Palacios M.A., Anzenbacher P. (2007). Rational design of a fluorescence-turn-on sensor array for phosphates in blood serum. Angew. Chem. Int. Ed..

[60] Kirby J.P., Cable M.L., Levine D.J., Gray H.B., Ponce A. (2008). Spectroscopic analysis of ligand binding to lanthanide-macrocycle platforms. Anal. Chem.

[61] Wu S.L., Horrocks W.D. (1996). General method for the determination of stability constants of lanthanide ion chelates by ligand-ligand competition: laser-excited Eu3+ luminescence excitation spectroscopy. Anal. Chem..

[62] Vaid A., Bishop A.H. (1998). The destruction by microwave radiation of bacterial endospores and amplification of the released DNA. J. Appl. Microbiol.

[63] Ashton P.R., Königer R., Stoddart J.F., Alker D., Harding V.D. (1996). Amino acid derivatives of β-cyclodextrin. J. Org. Chem.

[64] Hsu S.H., Yilmaz M.D., Blum C., Subramaniam V., Reinhoudt D.N., Velders A.H., Huskens J. (2009). Expression of sensitized Eu3+ luminescence at a multivalent interface. J. Am. Chem. Soc.

[65] Yuan J., Matsumoto K. (1996). Fluorescence enhancement by electron-withdrawing groups on-Diketones in Eu(lIl)-diketonato-topo Ternary Complexes. Anal. Sci.

[66] Gardeniers J.G.E., Oosterbroek R.E., van den Berg A., Oosterbroek R.E., van den Berg A. (2003). Silicon and glass micromachining for μTAS. Lab-on-A-Chip: Miniaturized Sytems for (Bio)Chemical Analysis and Synthesis.

[67] Wensink H., Elwenspoek M.C. (2002). A closer look at the ductile–brittle transition in solid particle erosion. Wear.

[68] Ludden M.J.W., Ling X.Y., Gang T., Bula W.P., Gardeniers H.J.G.E., Reinhoudt D.N., Huskens J. (2008). Molecular printboards inside microfluidic devices: small molecule and antibody recognition. Chem. Eur. J.

